# Single-fiber electromyography in the orbicularis oculi muscle in patients with ocular myasthenia gravis symptoms: does abnormal jitter predict response to treatment?

**DOI:** 10.1186/s12883-017-0891-5

**Published:** 2017-06-07

**Authors:** Goran Rakocevic, Mark Moster, Mary Kay Floeter

**Affiliations:** 1 Department of Neurology, Thomas Jefferson University, 901 Walnut Street, Room 408, Philadelphia, PA 19107 USA; 2 Wills Eye Hospital, Thomas Jefferson University, Philadelphia, PA USA; 3 EMG Section, National Institute of Neurological Disorders and Stroke, National Institutes of Health, Bethesda, MD USA

**Keywords:** Ptosis, Diplopia, Single-fiber EMG, Neuromuscular jitter, Acetylcholine receptor antibody

## Abstract

**Background:**

Seronegative ocular myasthenia gravis (OMG) is diagnosed by ocular symptoms with supporting SFEMG, typically of frontalis or extensor digitorum muscles. We aimed to determine the sensitivity and specificity of orbicularis oculi SFEMG to diagnose and exclude myasthenia gravis and predict response to therapy.

**Methods:**

Orbicularis oculi SFEMG studies were conducted in 142 consecutive patients with symptoms and/or findings of OMG and negative AChR antibody during the period of 5 years. Retrospective chart review was conducted 2 years after the SFEMG to determine whether treatments were given and responses to treatment.

**Results:**

Orbicularis oculi SFEMG was abnormal in 31 patients and normal in 111 patients. Twenty-nine patients with abnormal SFEMG were treated, and 25 had a good response. Twenty-four patients with normal SFEMG received treatment; none responded to treatment or developed generalized myasthenia.

**Conclusion:**

An abnormal orbicularis oculi SFEMG in patients with seronegative OMG has a high predictive value for response to therapy. Our study findings may affect the treatment decisions in practice and aid better management of myasthenic patients.

## Background

SFEMG is the most sensitive diagnostic test for diagnosis of seronegative myasthenia gravis [1–3]. Frontalis, orbicularis oculi and extensor digitorum communis (EDC) are commonly tested muscles in the electrophysiological exam. There are no current guidelines to apply when deciding what and how many muscles to evaluate in order to increase the yield of diagnostic sensitivity and specificity. Based on several studies comparing SFEMG of frontalis and or EDC muscles with repetitive nerve stimulation (RNS) [4–6], SFEMG is established as more sensitive test for diagnosing NMJ disorder than RNS. More recent studies investigated the utility of SFEMG in orbicularis oculi muscles in diagnosing MG. These studies, carried out on a mixed population of patients with focal or generalized myasthenic symptoms, found that SFEMG of the orbicularis oculi was reliable for diagnosis of MG [7] and had prognostic value for predicting the severity of patients’ clinical course [8]. We postulated that SFEMG in orbicularis oculi muscle would be particularly relevant for diagnosis and predicting response to therapy in a cohort of seronegative patients with typical visual symptoms of ocular myasthenia.

## Methods

Eighty-six women and fifty-six men with negative AChR antibody and fluctuating ocular symptoms (double vision in 79, ptosis in 37, and both in 51 patients) were referred for evaluation of ocular myasthenia gravis. The clinical diagnosis of ocular myasthenia (OMG) was based on the presence of at least one of cardinal visual symptom (diplopia) and/or findings on exam (variable extraocular muscle weakness and ptosis) by two examiners: a referring neuro-ophthalmologist and examining neurologist. All patients had ocular symptoms and/or findings for at least one month prior to SFEMG. Three patients were adolescents of 15, 16 and 17 years of age. A complete neuro-ophthalmologic evaluation including an MRI of brain with orbital protocol was performed in all patients to exclude other neurological disorders. All patients had laboratory testing for serum AchR and six were tested for anti-MuSK antibody. No patients had undergone genetic testing for other possible causes of external ophthalmoplegia prior to SFEMG, and during the follow up period after electrophysiological study.

SFEMG in orbicularis oculi muscle was intended as the measured variable, while the clinical criterion used as the gold standard was objective eyelid and/ or orbicularis oculi and extraocular muscle weakness on exam with fluctuating symptoms and findings.

SFEMG was performed on both orbicularis oculi muscles in all patients by the same examining neurologist in a single center (GR), using a SFEMG needle with standard techniques. A minimum of twenty pairs of muscle fibers were recorded from right and left orbicularis oculi muscle from all patients. The SFEMG study was considered abnormal: 1) if the mean jitter (MCD) in twenty pairs exceeded normal values for the orbicularis oculi and the patient’s age [7, 9]; or 2) if >10% of all studied pairs had jitter above the maximum allowed for a single pair using values adjusted for age.

Following the SFEMG study, treatment decisions were determined by referring physicians. Two-year follow up information for all patients was acquired by chart review, phone contact and office examination. The determination of generalized MG was based on development of symptomatic bulbar and/ or limb weakness confirmed by clinical examination by referring or treating neurologist. The medical charts were reviewed and patients’ symptoms and response to therapy, if any, were documented during a 2-year follow-up period. The determination about a therapeutic response and whether generalized myasthenia (GMG) developed were based on the examinations by neuro-ophthalmologists and treating neurologists in the clinic and documented in the medical records or by a telephone interview.

T-tests were used to compare demographic data of patients with normal SFEMG to those with abnormal SFEMG, with *p* < 0.05 as threshold for significance. A positive response to therapy was defined as objective improvement of symptoms and findings on exam. The relationship between a positive response and an abnormal SFEMG was calculated using Fisher’s exact test. The sensitivity and specificity of orbicularis oculi muscle SFEMG to predict treatment-responsive OMG and development of generalized MG were also calculated. Statistics were carried out in Prism 6 (www.graphpad.com).

## Results

One hundred and eleven patients had normal jitter and thirty-one had abnormal jitter. There was no significant difference in the mean age or disease duration of the two groups. Gender was equal in the group with abnormal jitter. Women made up 63% of the patients in the group with normal jitter.

Twenty-nine of 31 patients with abnormal jitter received treatment: pyridostigmine first, and if favorable but suboptimal response, by adding prednisone until resolution of symptoms. Two were not treated and 1 patient’s follow-up information was missing.

Twenty-five patients had a positive response to treatment as defined by improvement of ocular symptoms and signs, and based on objective neurologic or ophthalmologic exam (Table 1). In the group of patients with normal jitter, 24 patients were treated and 87 patients were not treated. Pyridostigmine alone and/or combined with oral steroids did not provide benefit in non-responders regardless of an underlying etiology, which was not conclusively established for each non-responder. More women than men with normal jitter were treated. This difference in treatment by gender was significant. None of the treated patients with normal jitter had a positive response to treatment. Thus, abnormal jitter was significantly associated with a beneficial response to treatment (Fisher exact test *p* < 0.001). Abnormal jitter had 100% sensitivity and 85.7% specificity for response to therapy. The positive predictive value was 86.2% and negative predictive value was 100% for response to treatment.

**Table 1:** Response to MG therapy and SFEMG findings in patients with fluctuating ocular symptoms

Data analyzed	No response	Responded	Total
Group with abnormal jitter	4	25	29
Group with normal jitter	24	0	24
Total	28	25	53

Four patients with negative MuSK antibody were given pyridostigmine trial with no benefit. Another patient was later found to have MuSK antibodies after a normal SFEMG, but did not receive any myasthenic therapy due to intercurrent cancer. A single patient who presented with ocular symptoms and had an abnormal SFEMG eventually developed generalized myasthenia.

## Discussion

This study confirms usefulness of SFEMG for detecting a NMJ disorder in patients with fluctuating extraocular muscle weakness and negative antibodies by recording SFEMG from orbicularis oculi muscles. Additionally, we have shown that abnormal jitter in orbicularis oculi muscles in patients presenting with a clinical picture of ocular myasthenia correlated with response to myasthenic therapies. The sensitivity and specificity of SFEMG was studied in this patient cohort retrospectively and correlated with their response to myasthenic therapy, if administered by referring physicians. An abnormal SFEMG was not predictive of subsequent development of generalized MG as only one of 29 patients with an abnormal SFEMG subsequently developed generalized MG. No patient with normal SFEMG responded to treatment or developed generalized MG. Our findings are in concordance with Rostedt et all retrospective study in EDC [10] and Padua’s prospective study [7] demonstrating SFEMG in orbicularis oculi muscle as the test of highest value in diagnosing ocular and generalized myasthenia.

## Conclusion

A high diagnostic sensitivity of SFEMG in orbicularis oculi muscle in identifying patients who are unlikely to respond to myasthenic therapies is the most objective diagnostic tool available to clinicians managing patients with fluctuating ocular symptoms and negative NMJ antibodies. The relevance of our study highlights importance of SFEMG in the treatment decisions and management of patients with ocular myasthenia.

## Abbreviations

AchR: Acetylcholine Receptor
CT: Computerized tomography
EDC: Extensor digitorum communis
GMG: General myasthenia gravis
MCD: Mean consecutive discharge
MRI: Magnetic resonance imaging
MuSK: Muscle-specific kinase
NMJ: Neuromuscular junction
OMG: Ocular myasthenia gravis
RNS: Repetitive stimulation study
SFEMG: Single fiber EMG
